# Mechanism of Action of Isopropoxy Benzene Guanidine against Multidrug-Resistant Pathogens

**DOI:** 10.1128/spectrum.03469-22

**Published:** 2022-12-08

**Authors:** Jie Li, Xiufeng Zhang, Ning Han, Peng Wan, Feifei Zhao, Tiantian Xu, Xianfeng Peng, Wenguang Xiong, Zhenling Zeng

**Affiliations:** a Guangdong Provincial Key Laboratory of Veterinary Pharmaceutics Development and Safety Evaluation, College of Veterinary Medicine, South China Agricultural University, Guangzhou, China; b National Laboratory of Safety Evaluation (Environmental Assessment) of Veterinary Drugs, South China Agricultural University, Guangzhou, China; c National Risk Assessment Laboratory for Antimicrobial Resistance of Animal Original Bacteria, South China Agricultural University, Guangzhou, China; d Guangzhou Insighter Biotechnology Co., Ltd., Guangzhou, China; Yangzhou University

**Keywords:** antibiotic resistance, isopropoxy benzene guanidine, multidrug-resistant pathogens, antibacterial activity, colistin

## Abstract

The increasing emergence of antibiotic resistance is an urgent threat to global health care; thus, there is a need for new therapeutics. Guanidine is the preferred functional group for antimicrobial design and development. Herein, the potential antibacterial activity of the guanidine derivative isopropoxy benzene guanidine (IBG) against multidrug-resistant (MDR) bacteria was discovered. The synergistic antibacterial activity of IBG and colistin was determined by checkerboard assay, time-killing curve, and mouse experiments. The antibacterial mechanism of IBG was verified in fluorescent probe experiments, intracellular oxidative phosphorylation assays, and transcriptome analysis. The results showed that IBG displays efficient antibacterial activity against Gram-positive pathogens and Gram-negative pathogens with permeabilized outer membranes. Further mechanistic studies showed that IBG triggers cytoplasmic membrane damage by binding to phosphatidylglycerol and cardiolipin, leading to the dissipation of proton motive force and accumulation of intracellular ATP. IBG combined with low levels of colistin enhances bacterial outer membrane permeability and increases the accumulation of reactive oxygen species, as further evidenced by transcriptome analysis. Furthermore, the efficacy of IBG with colistin against MDR Escherichia coli in three infection models was demonstrated. Together, these results suggest that IBG is a promising adjuvant of colistin, providing an alternative approach to address the prevalent infections caused by MDR Gram-negative pathogens.

**IMPORTANCE** As antibiotic discovery stagnates, the world is facing a growing menace from the emergence of bacteria that are resistant to almost all available antibiotics. The key to winning this race is to explore distinctive mechanisms of antibiotics. Thus, novel efficient antibacterial agents and alternative strategies are urgently required to fill the void in antibiotic development. Compared with the large amount of money and time required to develop new agents, the antibiotic adjuvant strategy is a promising approach to inhibit bacterial resistance and increase killing of bacteria. In this study, we found that the guanidine derivatives IBG not only displayed efficient antibacterial activities against Gram-positive bacteria but also restored colistin susceptibility of Gram-negative pathogens as an antibiotic adjuvant. More in-depth study showed that IBG is a potential lead to overcome antibiotic resistance, providing new insight into future antibiotic discovery and development.

## INTRODUCTION

Multidrug-resistant (MDR) and extensively drug-resistant (XDR) bacteria pose increasing threats to public health (1, 2), especially Gram-negative pathogens. The major challenge to treating infections caused by Gram-negative pathogens is that the outer membrane has low permeability (3–5). The influx of antibiotics is limited by the outer membrane, which restricts their access to intracellular targets (5). Increasing the number of available antibiotics is vital for addressing the growing resistance crisis (5, 6). However, the infrastructure for discovering and developing novel antibiotics has stagnated in recent years, and the rate of development of antibacterial drugs is far behind the speed of development of bacterial resistance (5, 7). The rates of mortality caused by untreatable infections are predicted to rise more than 10-fold by 2050 without new therapies (8). Considering the emerging resistance to traditional antibiotics coupled with the lack of novel antibiotics to combat antibiotic-resistant bacteria, alternative strategies are required to ease this crisis (9).

Drug combinations have emerged as a promising alternative approach to provide novel therapeutic options for MDR bacteria (7, 10). This strategy can overcome drug resistance by inhibiting multiple targets and minimizing further development of drug resistance (11, 12). Moreover, combination therapy with existing drugs significantly eases the crisis of new antibiotic vacancies and extends the life span of existing antibiotics (7, 13). For instance, the broad-spectrum antibiotic adjuvant SLAP-S25 can restore the efficiency of different classes of antibiotics against MDR Gram-negative bacteria (14). At present, the unexplored sources of new antibiotics or antibiotic adjuvants have been extended to various fields, such as natural compounds (15), marine organisms (16), chemical synthesis (14), and actinomycetes (17).

**FIG 1:**
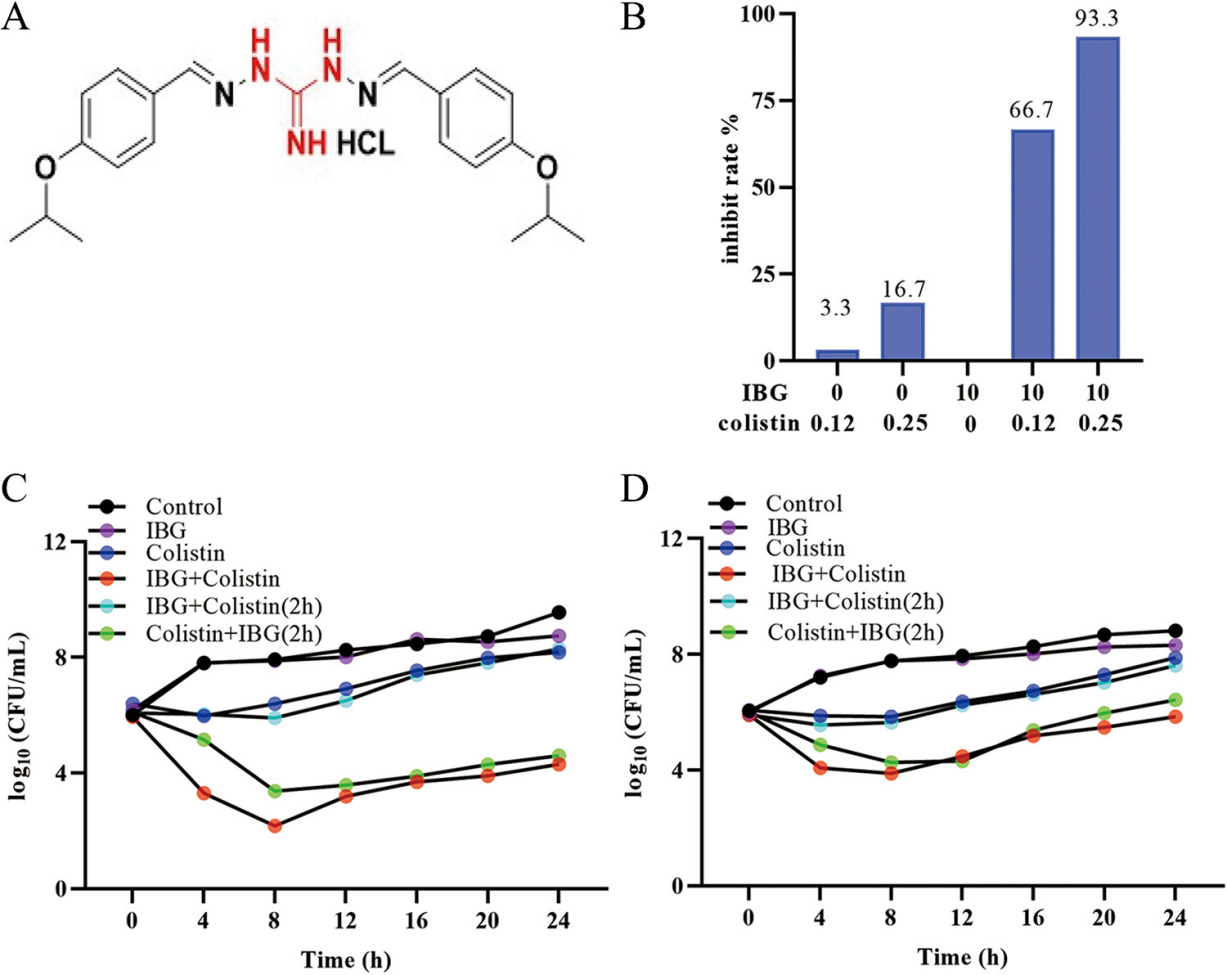
IBG restores the sensitivity of colistin against Gram-negative bacteria. (A) Chemical structure of the IBG. The guanidine group is marked in red. (B) Inhibition rate of colistin combined with IBG against 30 Gram-negative bacteria. (C and D) Time-kill assays conducted with colistin (0.5× MIC), IBG monotherapy (10 μg/mL), the combination, or later addition of colistin or IBG against colistin-susceptible E. coli ATCC 25922 (C) and colistin-resistant E. coli SHP45 (D).

Guanidine and guanidinium salts are frequently found in various natural sources (18). As a common functional group, guanidine has been identified in the design and development of antibacterial agents (19–21), and the guanidine group is also found in existing antibiotics, such as streptomycin, trimethoprim and chlorhexidine (22). At present, several guanidine compounds have been reported as promising antimicrobial agents, H-BDF (23) and NCL195 (24) included. Their antimicrobial activity depends on the presence of protonated guanidine groups and their resonance stabilization (22). We recently found that the guanidine compound isopropoxy benzene guanidine (IBG) (Fig. 1A) exerted a strong inhibitory activity against MDR Gram-positive bacteria, including methicillin-resistant Staphylococcus aureus and vancomycin-resistant enterococci (25, 26). Interestingly, in this study, we found that IBG combined with colistin exerted a synergistic antibacterial effect against Gram-negative bacteria, while IBG alone had no antibacterial activity. Further mechanistic studies indicated that IBG exerted multiple modes of antibacterial action. Notably, the efficacy of IBG potently rescued the activity of colistin, as observed in three infection models infected by MDR Escherichia coli. These results suggest that IBG is a potential compound for the design of membrane-active drugs.

## RESULTS

### IBG is a potential antibiotic adjuvant.

IBG alone had no activity (MIC > 2,560 μg/mL) against the Gram-negative bacteria tested. However, synergy was observed between IBG and colistin for all tested Gram-negative strains (Table 1). To further test whether this synergy is colistin specific, we assessed the activity of IBG in combination with different classes of antibiotics against E. coli ATCC 25922 and SHP45. Results indicated that IBG exhibited synergistic activity only with polymyxin B, which means that IBG is a potential adjuvant to polymyxins (see Table S1 in the supplemental material). Then, we tested the synergistic effect and found that the antibacterial activity of colistin increased 20-fold after addition of 10 μg/mL IBG, while only 3.3% (1/30) of the bacterial growth was inhibited after treatment with colistin alone. Moreover, the growth of more than 93% of the bacteria (28/30) was inhibited after treatment with 10 μg/mL IBG and 0.25 μg/mL colistin (Fig. 1B; Table S2). This potentiation seems comparable to that of recently reported compounds, including α-mangostin (15), isobavachalcone (15), pentamidine (27), and SLAP-S25 (14).

**TABLE 1:** Antibacterial activity of IBG^*a*^

Strain	Source	*mcr-1*	MIC (μg/mL)		FICI	Effect
			Colistin	IBG		
Gram-positive bacteria						
S. aureus ATCC 29213	Reference strain	−		4		
E. faecalis ATCC 29212	Reference strain	−		4		
Gram-negative bacteria						
Escherichia coli						
ATCC 25922	Reference strain	−	0.5	>2,560	0.12	Synergy
GDQ8D43	Pig	+	2	>2,560	0.12	Synergy
SHPP45	Pig	+	4	>2,560	0.12	Synergy
GDQ8P37	Pig	+	4	>2,560	0.06	Synergy
Salmonella						
ATCC 14028	Reference strain	−	0.5	>2,560	0.12	Synergy
S226	Chicken	+	2	>2,560	0.06	Synergy
S235	Pig	+	2	>2,560	0.12	Synergy
26FS14	Pig	+	2	>2,560	0.25	Synergy
Klebsiella pneumoniae						
ATCC 700603	Reference strain	−	1	>2,560	0.25	Synergy
MPC11	Pig	−	0.5	>2,560	0.25	Synergy
MPC11+ pHNSHP45	Pig	+	8	>2,560	0.12	Synergy
117	Pig	+	16	>2,560	0.25	Synergy
281	Pig	+	16	>2,560	0.25	Synergy
Acinetobacter baumannii						
ATCC 19606	Reference strain	−	1	>2,560	0.25	Synergy
130939	Human	−	2	>2,560	0.12	Synergy
131284	Human	−	4	>2,560	0.12	Synergy
Pasteurella multocida						
399	CVCC	−	2	>2,560	0.06	Synergy
434	CVCC	−	4	>2,560	0.03	Synergy
89	Pig	−	0.5	>2,560	0.06	Synergy
23	Pig	−	0.5	>2,560	0.03	Synergy

a ATCC, American Type Culture Collection; CVCC, China Veterinary Culture Collection Center; FICI, fractional inhibitory concentration index, observed in three independent experiments.

Furthermore, the time-killing experiment results showed that the combination of IBG and colistin displayed obvious bactericidal activity against E. coli ATCC 25922 and E. coli SHP45 (Fig. 1C and D). Interestingly, we found the antibacterial effect of adding IBG to the bacteria treated with colistin was stronger than that of adding colistin to the bacteria treated with IBG. Toxicity effect is a key factor that limits the combination therapy in the clinic, and the result showed that the addition of IBG had no obvious effect on the hemolysis of colistin (Fig. S1A).

### IBG inhibits the resistance development of colistin.

**FIG 2:**
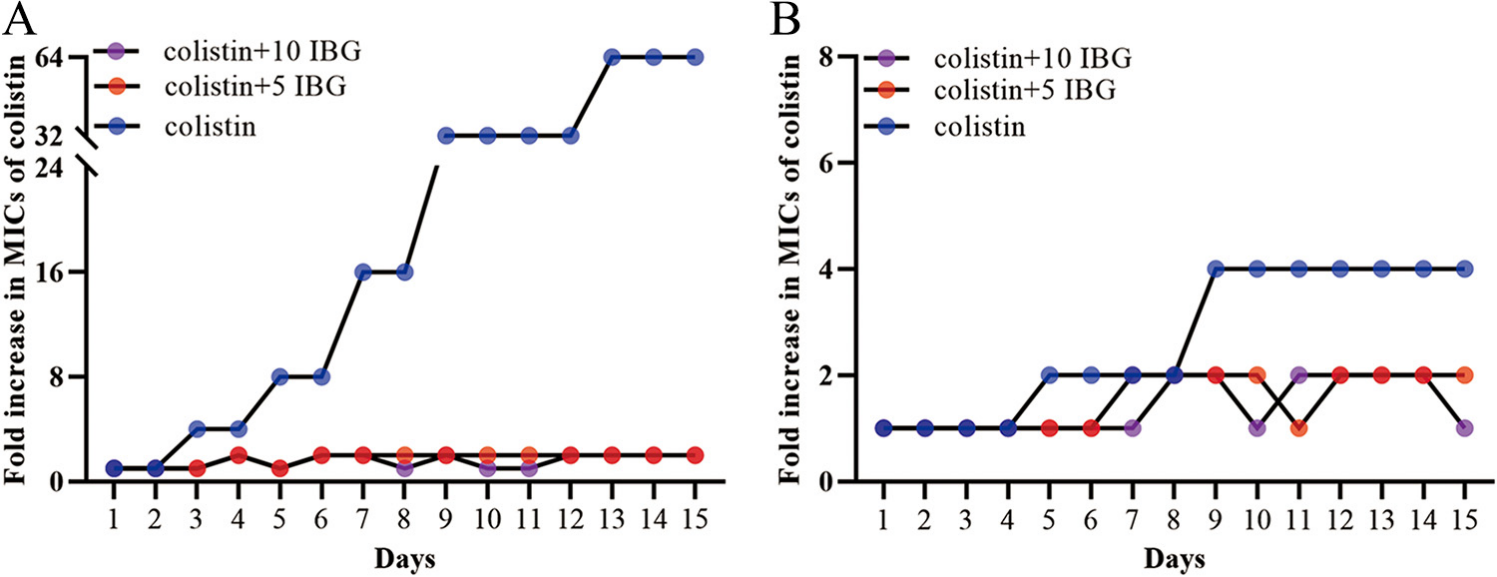
Evolution of colistin resistance in colistin-susceptible and colistin-resistant E. coli. The fold change of colistin MIC was detected after colistin-susceptible E. coli ATCC 25922 (A) and colistin-resistant E. coli SHP45 (B) were treated with 0.5× MIC colistin alone or in combination with IBG at 5 μg/mL or 10 μg/mL *in vitro*.

Severe resistance is one of the important reasons for clinical treatment failure; thus, the development of colistin resistance was also assessed in serial passages. We found that colistin monotherapy produced highly resistant strains with a 64-fold increase in the MIC for ATCC 25922 and strains with a 4-fold increase in the MIC for SHP45. Consistent with previous studies, colistin-resistant strains were less likely to increase the MIC of colistin than colistin-susceptible strains in a serial-passage experiment (28, 29). Only a 2-fold increase in the MIC of colistin was observed for both strains when they were treated with the combination (Fig. 2A and B). Compared with colistin alone, the addition of IBG inhibited the development of colistin resistance.

### IBG targets bacterial cytoplasmic membrane against MDR bacteria.

**FIG 3:**
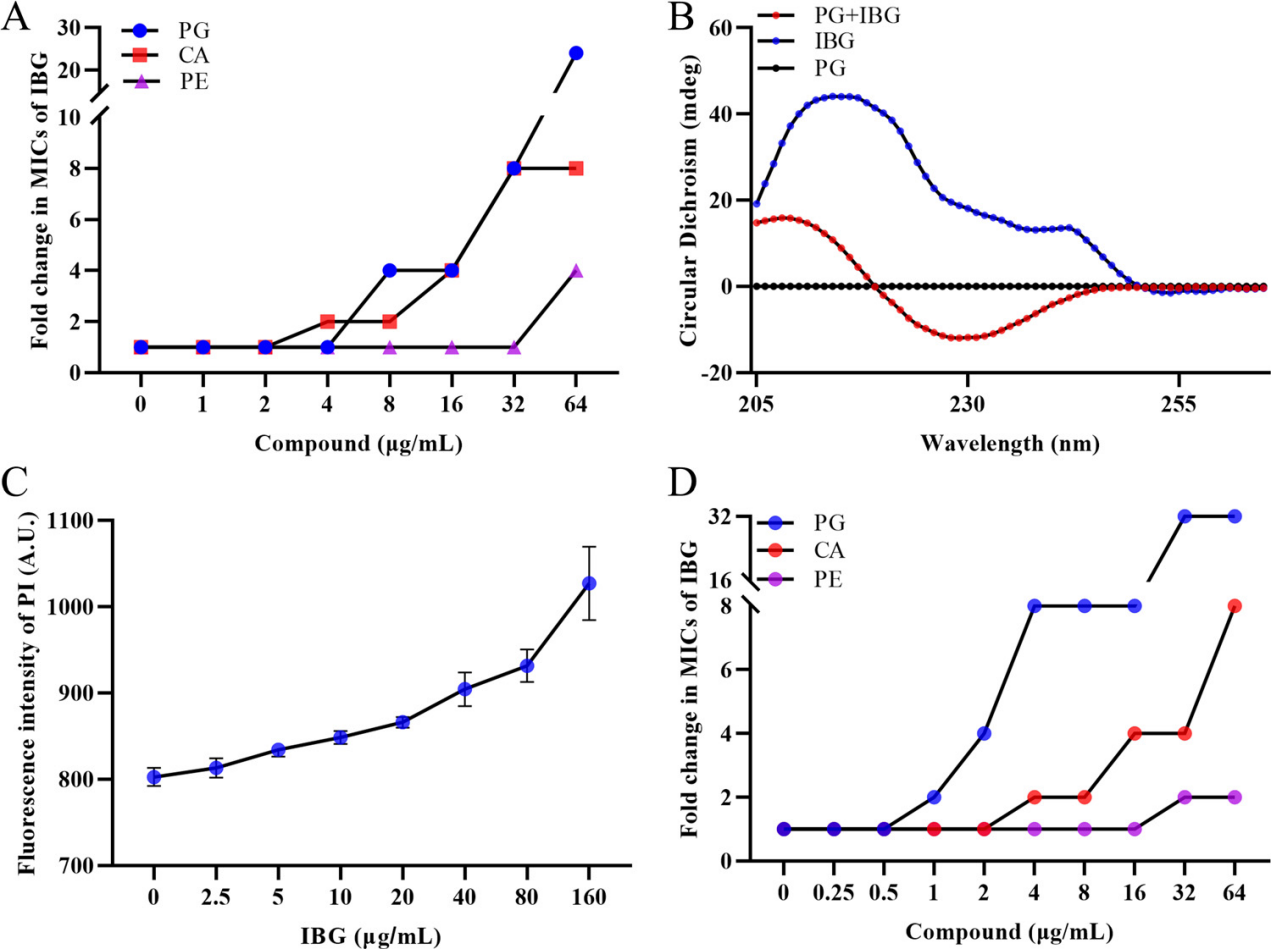
IBG exerts bactericidal activity by interacting with cytoplasmic membrane. (A) The antibacterial activity of IBG combined with phosphatidylglycerol (PG), phosphatidylethanolamine (PE), or cardiolipin (CA) was determined by checkerboard broth microdilution assays. (B) The conformational structure of IBG (10 μg/mL) with or without PG (32 μg/mL) was evaluated by the circular dichroism (CD) spectra. (C) Increased permeability of inner membrane was probed with PI for S. aureus ATCC 29213 treated with different concentrations of IBG. (D) Synergistic activity between IBG and three major membrane phospholipids against the LPS mutant strain MG1655-Δ*waaC* was detected, and PG and CA remarkably reduced the antibacterial activity of IBG.

Next, we evaluated the effects of the main components of the bacterial wall and cytoplasmic membrane (CM) on IBG activities against S. aureus ATCC 29213 by exogenous addition. Compared with the slight effect of peptidoglycan (Fig. S1B), a dose-dependent inhibition of IBG activity was observed with the exogenous addition of bacterial phospholipids, including phosphatidylglycerol (PG) and cardiolipin (CA), especially for PG (Fig. 3A). Meanwhile, the conformational structure of IBG could be changed in the presence of PG via circular dichroism (CD) spectroscopy (Fig. 3B), confirming that IBG has high affinity for PG. In addition, molecular docking showed that IBG has a high affinity for PgsA, which is an important protein in the synthesis of PG (30), with a CDocker interaction energy value of 22.0761 kcal/mol (Fig. S1C and D). As expected, the membrane permeability of S. aureus ATCC 29213 increased in a dose-dependent manner upon treatment with IBG, as determined by monitoring the uptake of propidium iodide (PI) (Fig. 3C).

**FIG 4:**
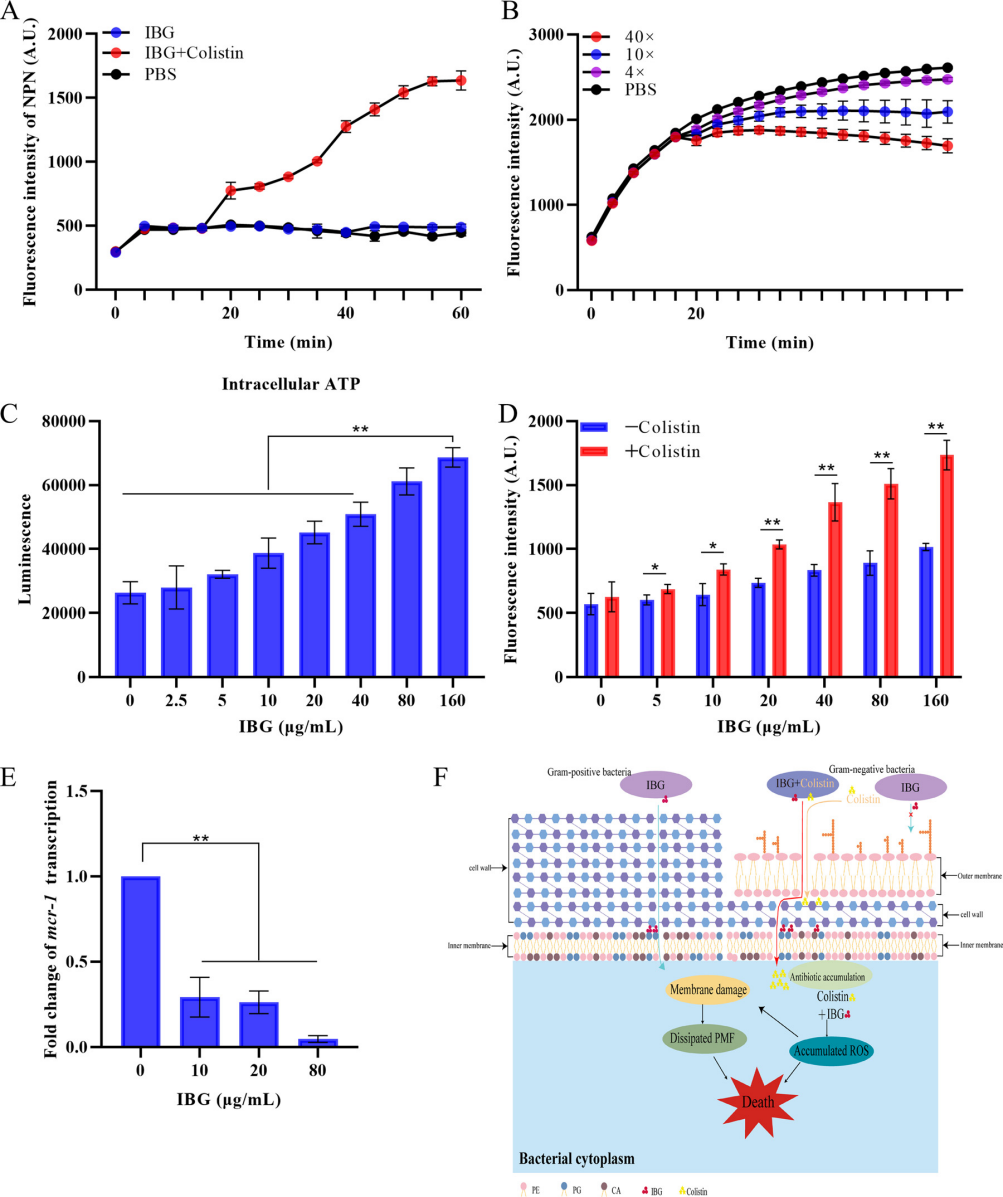
Antibacterial mechanism of IBG against MDR bacteria. (A) Dynamic curves of the outer membrane probed with NPN in E. coli ATCC 25922 treated with IBG (10 μg/mL), colistin (0.12 μg/mL), and both together. The fluorescence was detected with excitation and emission wavelengths of 350 nm and 420 nm. (B) The ΔpH after treatment with different concentrations of IBG (from 0 to 10× MIC) was determined in S. aureus ATCC 29213. The means for three biological replicates are shown, and error bars represent the SD. (C) Levels of intracellular ATP in S. aureus ATCC 29213 after treatment of IBG. Nonparametric one-way analysis of variance (ANOVA) was used to calculate *P* values (*, *P* < 0.05; **, *P* < 0.01). (D) Accumulation of ROS in E. coli ATCC 25922 treated with IBG with or without 0.12 μg/mL colistin. (E) IBG inhibits the transcript level of *mcr*-1 in E. coli SHP45 determined by qRT-PCR. All data are means and SD, and significance was determined by nonparametric one-way ANOVA (**, *P* < 0.01). (F) Scheme of mechanisms of action of IBG in Gram-positive and Gram-negative bacteria.

Considering that components of phospholipids in the bacterial CM of Gram-positive and Gram-negative bacteria are the same (31), we speculated that the bacterial outer membrane (OM) of Gram-negative bacteria acted as a permeability barrier to IBG reaching the phospholipids in Gram-negative bacteria. Accordingly, we assessed the effects of lipopolysaccharide (LPS) and divalent cations on the antibacterial activity of IBG, as LPS is a main component of the OM and divalent cations are main components and maintain the stability of the bacterial OM (5, 32). As expected, the exogenous addition of LPS and divalent cations had a negligible influence on the activity of IBG (Fig. S1E and F). In addition, the membrane permeabilizers EDTA and polymyxin B nonapeptide (PMBN) enhanced the antibacterial activity of IBG against Gram-negative bacteria (Fig. S1G and H). Hence, LPS deletion strains (MG1655-Δ*waaC* and MG1655-Δ*waaP*) were constructed using CRISPR-Cas9 (Fig. S2A and B), which disrupts the integrity of the OM. The MIC of IBG decreased by more than 64-fold for LPS deletion strains (MG1655-Δ*waaC* and MG1655-Δ*waaP*) compared with the wild-type E. coli MG1655 (Table S3). Meanwhile, the exogenous addition of PG and CA also reduced the activity of IBG against LPS deletion strains (Fig. 3D). To further validate of these findings, hydrophobic fluorescent probe 1-*N*-phenylnaphthylamine (NPN) was used to evaluate the damaging effect on the OM. We found that IBG alone had a negligible effect on the permeability of OM, whereas the IBG with only 0.12 μg/mL colistin rapidly increased the OM permeability (Fig. 4A). In addition, the intracellular accumulation of colistin was increased in the presence of IBG (Fig. S1I; Table S4), as determined by liquid chromatography-tandem mass spectrometry (LC-MS/MS) analysis.

### Mechanism of IBG against MDR bacteria.

Considering that IBG can disrupt the cell membrane potential (ΔΨ) and bacterial structure of S. aureus (Fig. S3A and B), we further determined the effect of IBG on ΔpH, the other component of the proton motive force (PMF), and we observed that IBG considerably dissipated the ΔpH in a dose-dependent manner (Fig. 4B). Meanwhile, we found synergy of kanamycin in combination with IBG, which is consistent with ΔΨ dissipation. In contrast, IBG showed no synergy with tetracycline (Fig. S3C). The disruption of PMF can interfere with the levels of cellular ATP (33, 34). Correspondingly, IBG significantly increased the intracellular ATP levels in a dose-dependent manner (*P* < 0.01) (Fig. 4C; Fig. S3D). The accumulation of reactive oxygen species (ROS) is a common mechanism caused by bactericidal antibiotics (35–37). Interestingly, the bactericidal mechanism of IBG alone is independent of ROS, whereas IBG significantly promotes the accumulation of ROS in the presence of low concentrations of colistin (0.12 μg/mL) in E. coli ATCC 25922 (*P* < 0.05) (Fig. 4D; Fig. S3E).

**FIG 5:**
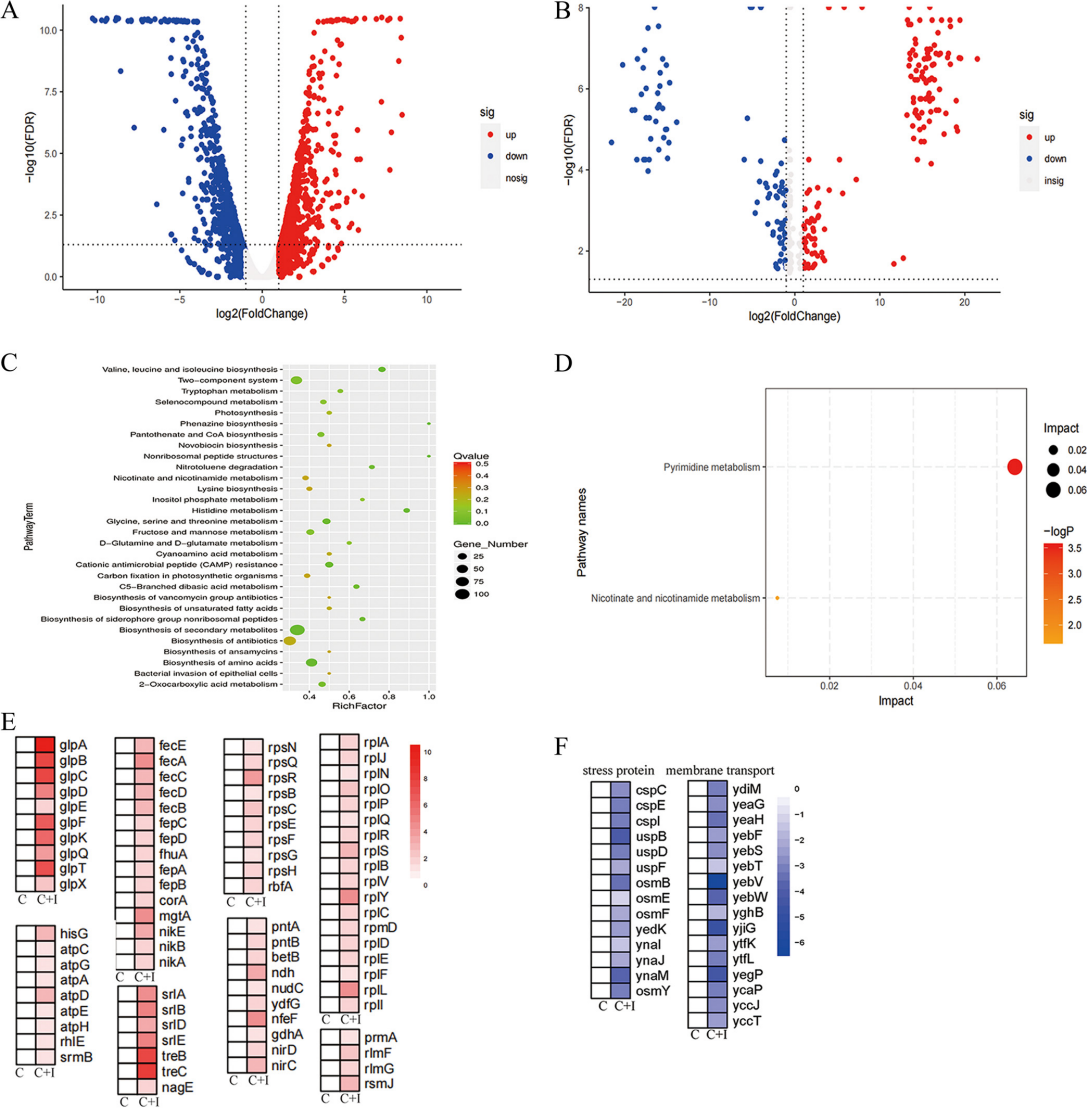
Transcriptome and metabolome analysis of E. coli after exposure to colistin alone or IBG-colistin. (A and B) Volcano plot analysis of DEGs (A) and DEMs (B) in E. coli ATCC 25922 after exposure to colistin or the combination of colistin and IBG for 4 h. (C and D) KEGG enrichment analysis of DEGs (C) and DEMs (D) after treatment with colistin or the combination. (E and F) Heat map of upregulated DEGs (E) and downregulated DEGs (F) in E. coli after exposure to colistin alone or IBG-colistin. An adjusted *P* value of <0.05 (Student's *t* test with Benjamini-Hochberg false discovery rate adjustment) and a |log_2_ fold change| of ≥1 were applied as the cutoffs for significant DEGs. C, colistin alone; C+I, combination of colistin and IBG.

Notably, IBG also inhibited the transcription of *mcr-1* gene in a dose-dependent manner (*P* < 0.01) (Fig. 4E). To further decipher the molecular mechanisms of IBG, we performed transcriptomic and metabonomic analysis of E. coli ATCC 25922 after exposure to colistin or colistin-IBG for 4 h. The comparison of treatment with the combination and that with colistin alone revealed 1,193 differentially expressed genes (DEGs) and 267 differentially expressed metabolites (DEMs) (Fig. 5A and B). KEGG (Kyoto Encyclopedia of Genes and Genomes) enrichment analysis demonstrated that these DEGs were significantly enriched in the biosynthesis of secondary metabolisms, antibiotics, and amino acids (Fig. 5C). Similarly, metabolite enrichment analysis revealed that pyrimidine metabolism, nicotinate, and nicotinamide metabolism were significantly affected in E. coli (Fig. 5D). Notably, we found an increase in the expression of genes involved in protein transport, including ribosomal transport, iron transport, energy and sugar import, and glycerol metabolism (Fig. 5E). Meanwhile, genes related to osmotic stress and membrane transport were significantly downregulated in the combination group (Fig. 5F). It is plausible that the combination caused the permeability of the bacterial membrane. In summary, our findings suggested that IBG exerts an antibacterial effect on Gram-positive bacteria by targeting bacterial phospholipids while potentiating colistin activity by promoting oxidative damage (Fig. 4F).

### *In vivo* efficacy.

**FIG 6:**
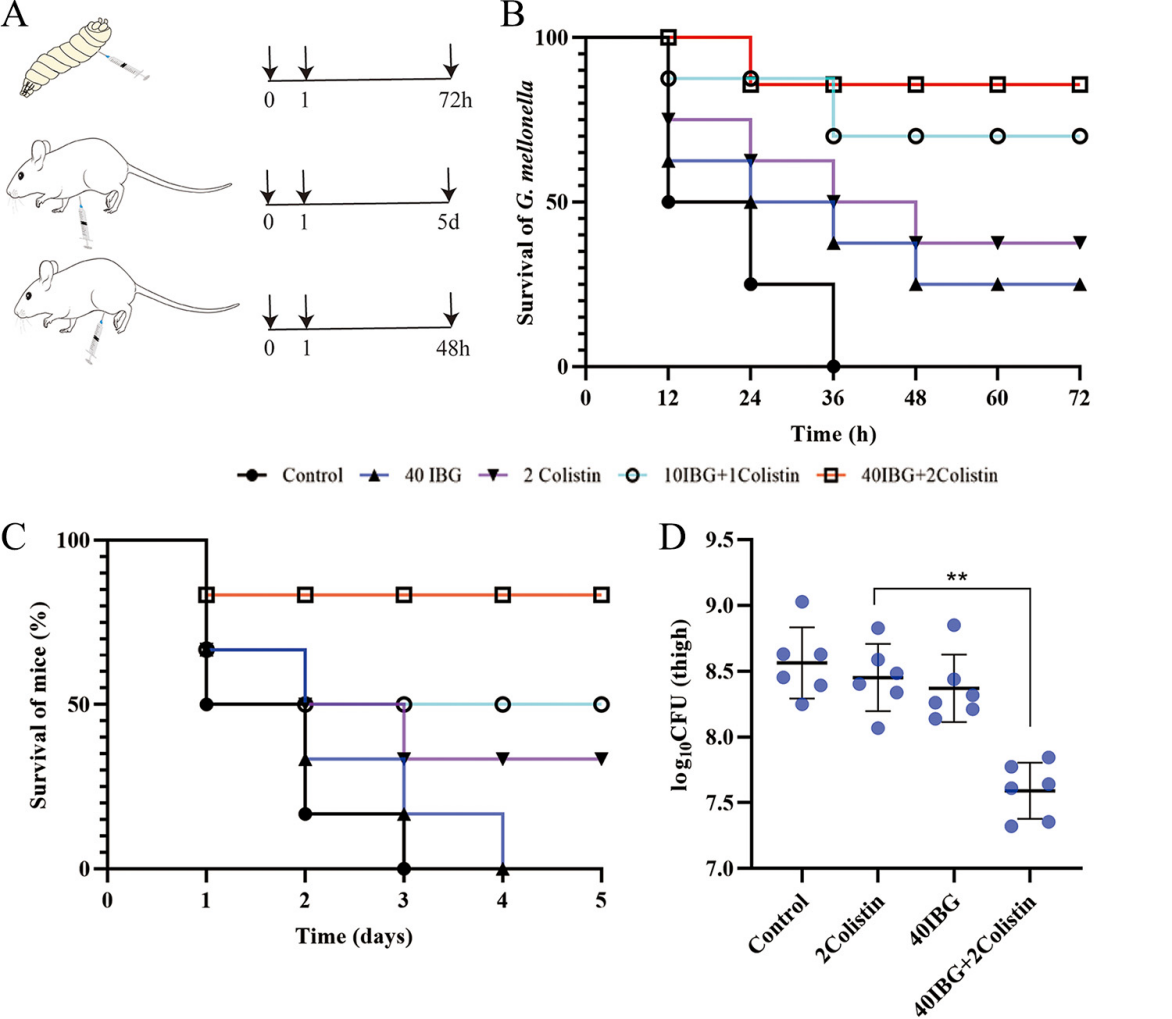
Efficacy of IBG combined with colistin *in vivo*. (A) Scheme of the experimental protocol for three animal models. (B) Survival rates of the G. mellonella larvae (10 per group) infected by E. coli SHP45 undergoing treatment with IBG (40 μg/mL) or colistin (2 μg/mL) alone or in combination (10 μg/mL IBG + 1 μg/mL colistin or 40 μg/mL IBG + 2 μg/mL colistin). (C) Survival rates of female BALB/c mice (6 per group) infected by E. coli SHP45 in the mouse peritonitis-sepsis model treated with a single dose of IBG (40 μg/mL) or colistin (2 μg/mL) alone or with IBG plus colistin (10 μg/mL + 1 μg/mL or 40 μg/mL + 2 μg/mL). (D) Bacterial loads in infected thigh muscle in mice (6 per group). *P* values were determined using nonparametric one-way ANOVA. Data are means and SD.

Given the attractive potentiation of IBG *in vitro*, a Galleria mellonella model and two mouse infection models were evaluated for the potential of IBG combination therapy against E. coli SHP45 *in vivo* (Fig. 6A). At 3 days after infection, the combination of IBG (40 mg/kg of body weight) and colistin (2 mg/kg) increased the survival of G. mellonella larvae (Fig. 6B). The efficacy of colistin combined with IBG was also tested in a mouse peritonitis-sepsis model. Compared to monotherapy, IBG-colistin effectively protected mice from infection by colistin-resistant and colistin-sensitive strains (Fig. 6C; Fig. S1J). Consistently, the number of inflammatory cells significantly decreased, and typical pathological changes around the infected organs were greatly alleviated as well (Fig. S4). Furthermore, the bacterial counts in a mouse thigh infection model decreased significantly upon treatment with the combination (*P* < 0.01) (Fig. 6D).

## DISCUSSION

The emergence and rapid spread of antibiotic resistance in pathogenic bacteria pose a severe threat to public health worldwide (7, 38). Alarmingly, the spread of the plasmid-borne colistin-resistant *mcr* gene results in difficulty in treatment of infections caused by Gram-negative bacteria, and colistin is considered a last-resort antibiotic for Gram-negative pathogens resistant to essentially all other antibiotics (39–41). However, its clinical use is limited due to its adverse effects, particularly the potential for nephrotoxicity and neurotoxicity, (42, 43). In the present study, 10 μg/mL IBG enhanced the antibacterial activity of colistin, thus reducing the dosage and toxic side effects of colistin.

Interestingly, IBG exhibited completely different antibacterial effects in Gram-positive bacteria and Gram-negative bacteria. Based on the difference in membrane structures (15, 16), we speculate that the difference of membrane is the main reason. Moreover, IBG restored antibacterial activity against Gram-negative bacteria in the presence of membrane permeabilizers, revealing that OM is a physical barrier. The functions and physical integrity of the CM are critical (44). Disruption of bacterial CM may cause intracellular metabolic disturbances. ROS are generally considered to cause a variety of types of intracellular damage, including damaging DNA, peroxidating lipids, and carbonylating proteins (37, 45). The accumulation of intracellular ROS amplifies this damage and may be the terminal step leading to bacterial death (37). We found that IBG promoted the accumulation of colistin, which in turn led to the accumulation of ROS. Subsequently, the accumulation of ROS triggered ROS-mediated membrane damage, which may account for the synergy between IBG and colistin. Increased respiration is associated with antimicrobial activity (46–48). Consistent with previous findings, IBG alone can accelerate the accumulation of intracellular ATP. Accordingly, the results were also confirmed in the transcriptome and metabolome. Specifically, the upregulation of 30S and 50S subunit-synthetic genes in combination may be caused by the intracellular accumulation of colistin in E. coli and inhibited protein synthesis, which is compensated by an upregulation of ribosome synthesis-related genes (42). These results agreed with the intracellular accumulation of colistin in the presence of IBG (Fig. S1I). Oxidative-phosphorylation-related genes, such as ATP synthase and NADH-quinone oxidoreductase genes, were significantly upregulated, thus supporting the increased ATP level under combination treatment (35, 47, 49).

The binding of IBG to the cytoplasmic membrane is its main mode of action with regard to the cytoplasmic membrane. Exogenous addition of PG and CA, major cytoplasmic membrane components, effectively inhibits the antibacterial activity of IBG, supporting the idea that IBG acts as a PG- and CA-targeting antibiotic. Furthermore, IBG may target the same components in both Gram-positive and Gram-negative bacteria, because the antibacterial activity of IBG was decreased in LPS-deficient bacteria by exogenous addition of PG and CA. Compared with traditional antibiotics, membrane-targeting antibiotics can effectively reduce the emergence of resistance (44). We are surprised to find that the addition of only 10 μg/mL IBG can inhibit the emergence of colistin resistance after serial passage, which is of great significance for prolonging the clinical life of colistin. Moreover, synergistic drug combinations are used for the treatment of severe infections caused by drug-resistant bacteria *in vivo* (7, 50). The therapeutic effect of IBG combined with colistin was significantly improved compared with that of single-drug treatment.

In conclusion, IBG exhibited good antibacterial activities against Gram-positive bacteria. At the same time, IBG, serving as an antibiotic adjuvant, acted synergistically with colistin against diverse MDR Gram-negative bacteria (including colistin-resistant and -susceptible strains) and potentiated the therapeutic effect of colistin *in vivo*. IBG is a potential lead compound for the design of membrane-active drugs. Although the therapeutic effect has been confirmed *in vivo*, the pharmacokinetics and pharmacodynamics of IBG need to be further optimized. Although the mechanism of IBG has been demonstrated initially, the specific binding target of IBG is currently unclear. Taken together, these observations show that further studies are needed to explore the specific targets for membrane-associated genes.

## MATERIALS AND METHODS

### Strains and plasmids.

All strains were performed at 37°C with shaking at 180 rpm, and all bacterial strains used are listed in Table S5. Plasmid pEcgRNA/pEcCas was used for genome editing.

### Antimicrobial susceptibility test.

The MICs of IBG, EDTA, PMBN, and different antibiotics were determined by the standard broth microdilution method according to the Clinical and Laboratory Standards Institute (51) guideline. The bacteria overnight cultures were adjusted to a 0.5 McFarland turbidity standard. Then, antibiotics were 2-fold serially diluted in Mueller-Hinton broth (MHB) and mixed with equal volumes of bacterial inoculum in a clear UV-sterilized 96-well microtiter plate. After 18 h of incubation at 37°C, the MICs resulting in complete growth inhibition for each tested agent were determined by visual inspection.

### Checkerboard analyses.

The synergistic activity between antibiotics and melatonin was evaluated by checkerboard assays with 2-fold serially diluted concentrations of drugs (eight-by-eight matrix), and three independent experiments were used to calculate the fractional inhibitory concentration indexes (FICIs). FICIs were calculated according to the formula FICI_a_ + FICI_b_, calculated as (MIC_ab_/MIC_a_) + (MIC_ba_/MIC_b_), where MIC_a_ is the MIC of compound A alone, MIC_ab_ is the MIC of compound A in combination with compound B, MIC_b_ is the MIC of compound B alone, and MIC_ba_ is the MIC of compound B in combination with compound A. Synergy is defined as a FICI of <0.5, addition as 0.5 ≤ FICI ≤ 1, indifference as 1 < FICI ≤ 4, and antagonism as a FICI of >4.

### Hemolytic assay.

The hemolytic activity of IBG and colistin was evaluated using sterile defibrinated sheep blood cells. First, sheep blood cells were collected and washed with 0.9% sterile saline. Then, after treatment with different concentrations of colistin (0, 1, or 2 μg/mL) with or without IBG (0, 5, 10, or 40 μg/mL), 0.2% Triton X-100 and phosphate-buffered saline (PBS) were used as positive and negative controls, respectively. After 1 h, the supernatant was obtained for measurement at 576 nm, and the hemolysis rate was calculated as a percentage, as follows: [(OD_sample_ − OD_negative control_)/(OD_positive control_ − OD_negative control_) × 100], where OD is optical density.

### Emergence of resistance assay.

The cultures were serially passaged for 15 days, and development of resistance to IBG monotherapy or colistin-IBG combination therapy was determined. Overnight cultures of E. coli ATCC 25922 and E. coli SHP45 were diluted in fresh MHB containing colistin or colistin plus a fixed concentration of IBG (5 or 10 μg/mL). The bacterial cells were harvested after 18 h of incubation at 37°C, and a MIC assay was then performed using 96-well microtiter plates. The fold change in the MIC of colistin relative to the initial MIC was calculated.

### Time-kill assays.

The overnight cultures of colistin-resistant strain (E. coli SHP45 with the *mcr-1* gene) and colistin-susceptible strain (E. coli ATCC 25922) were diluted 1:100 (about 1 × 10^6^ CFU/mL) and treated with 0.5× MIC of colistin, IBG (10 μg/mL) alone, or the combination. Moreover, under some conditions, IBG was added after 2 h of colistin treatment or colistin was added after 2 h of IBG treatment. Colony counts were obtained in duplicate at 0, 4, 8, 12, 16, 20, and 24 h. Synergy was defined as a ≥2-log_10_-CFU/mL reduction between the combination and the most active single agent at 24 h.

### Scanning electron microscopy analysis.

The morphological appearance of S. aureus ATCC 29213 was observed by scanning electron microscopy (SEM). In brief, bacterial cultures of ATCC 29213 were collected and resuspended in PBS. After being treated with different concentrations of IBG (0, 1× MIC, 10× MIC) for 4 h, bacterial cultures were collected and fixed in a 2.5% glutaraldehyde solution. The structure of S. aureus was observed under a Hitachi SU 8010 SEM (Hitachi High Technologies, Tokyo, Japan).

### Antibacterial activity of the exogenous addition.

To understand the effect of exogenous addition on antibacterial activity of IBG against S. aureus ATCC 29213 and E. coli MG 1655-Δ*waaC*, levels of PG (Sigma-Aldrich; ≥99%), PE (Sigma-Aldrich; ≥99%), CA (Sigma-Aldrich; ≥99%), peptidoglycan, LPS, and different cations (NaCl, CaCl_2_, and MgCl_2_; Aladdin) were evaluated using checkerboard assays.

### CD analysis.

The CD spectral signature of IBG in the absence or presence of colistin was evaluated by CD spectroscopy. In brief, samples of IBG (10 μg/mL), PG alone (32 μg/mL), and the combination of IBG and colistin were prepared. The scan wavelength ranged from 205 nm to 265 nm, and the interaction between IBG and PG was analyzed according to the unique spectrum.

### Antibiotic accumulation analysis.

The accumulation of colistin in E. coli SHP45 was determined based on LC-MS/MS analysis. Briefly, bacteria were treated with colistin in the presence and absence of IBG. To lyse the samples, we filtered three freeze-thaw cycles in liquid nitrogen, and the resulting supernatants were filtered through 0.2-μm-pore-size filters. The filtered supernatants were diluted 10-fold and subjected to LC-MS/MS quantification.

### LPS mutations induced by CRISPR-Cas9.

A CRISPR Cas9 two-plasmid system (pEcgRNA/pEcCas) was used for genome editing in E. coli MG1655 strains according to the previous report (52). Twenty nucleotides of target-specific sequences was selected and annealed to form double-strand DNA (dsDNA) and then ligated to BsaI-linearized pEcgRNA to generate the new target-specific plasmid pEcgRNA-T. Competent cells of E. coli MG1655 carrying pEcCas were prepared. Then, the cells were mixed with plasmid pEcgRNA-T, and the mixture was transferred into a 2-mm electroporation cuvette (Bio-Rad) and electroporated at 1.8 kV, 200 Ω, and 25 μF. The cells were recovered in 1 mL antibiotic-free LB broth and incubated at 37°C for 1.5 h before being spread on LB plates containing kanamycin (50 μg/mL) and spectinomycin (50 μg/mL). After electroporation and recovery of transformants on selection plates, individual colonies were randomly picked and verified by colony PCR. Sanger sequencing was used for all PCR products. The E. coli strains MG1655-Δ*waaC* and MG1655-Δ*waaP* were constructed, and the primers are shown in Table S6.

### Permeabilization assay of OM to NPN.

NPN uptake assays were used to evaluate the OM integrity of E. coli ATCC 25922 treated with IBG alone or combined with colistin. Briefly, a single colony was grown overnight and centrifuged at 5,000 rpm for 10 min. Then, the cells were washed and resuspended in 5 mM HEPES (pH 7.2, 5 mM glucose). NPN was added to a final concentration of 10 μM, and the mixture was incubated at 37°C. A total of 10 μL of different concentrations of drugs was added to the mixture, and fluorescence was measured with an excitation wavelength of 350 nm and an emission wavelength of 420 nm with an EnSight multimode plate reader.

### Membrane integrity test.

The membrane permeability of E. coli ATCC 25922 induced by IBG was tested with the fluorescent dye propidium iodide (PI) (Aladdin, China) at a final concentration of 10 nM. Overnight cultures of E. coli ATCC 25922 cells were washed three times with 5 mM HEPES containing 20 mM glucose (pH 7.2), and bacterial suspensions were adjusted to an OD_600_ nm of approximately 0.5. After incubation with PI at 37°C for 20 min, the S. aureus cells were treated with IBG, and fluorescence was measured with an excitation wavelength of 535 nm and an emission wavelength of 615 nm.

### Membrane potential assay.

The membrane potential (ΔΨm) of S. aureus ATCC 29213 induced by IBG was tested by 3,3′-dipropylthiadicarbocyanine iodide [DiSC_3_(5); Thermo Scientific, United States]. In brief, S. aureus ATCC 29213 cells were washed three times with 5 mM HEPES containing 20 mM glucose at pH 7.2 and then incubated with 1 mM DiSC_3_(5) at 37°C for 20 min. The bacterial cells were treated with different concentrations of IBG. Subsequently, the fluorescence of the DiSC_3_(5) dye was monitored every 5 min for 60 min at 622-nm excitation and 670-nm emission.

### Molecular docking study.

The model structure of PG synthesis-related protein (PgsA) was found in the UniProt Knowledgebase. The two-dimensional (2D) structure of IBG was drawn using ChemDraw software. The target protein-ligand linking process was assessed and visualized using the CDocker protocol of Discovery Studio.

### ΔpH assay.

The ΔpH of S. aureus ATCC 29213 was determined by the pH-sensitive fluorescence probe BCECF-AM [2′,7′-bis(2-carboxyethyl)-5(6)-carboxyfluorescein acetoxymethyl ester]. Cells cultured overnight were washed and resuspended with HEPES (5 mM [pH 7.0]; plus 5 mM glucose) to obtain an OD_600_ of 0.5. BCECF-AM was added, and the mixture was incubated at 37°C. After 20 min, different concentrations of IBG were added, and the fluorescence value was determined at an excitation wavelength of 488 nm and emission wavelength of 535 nm.

### ATP determination.

The ATP levels of S. aureus ATCC 29213 were determined using an Enhanced ATP assay kit (Beyotime, China). Overnight cultures of S. aureus ATCC 29213 were washed three times with 0.01 M PBS (pH 7.4) and resuspended to obtain an OD_600_ of 0.5. After treatment with various concentrations of IBG, ranging from 0 to160 μg/mL, at 37°C for 60 min, bacterial cultures were centrifuged at 12,000 rpm for 5 min, and the supernatant was collected for the determination of extracellular ATP level, while the bacterial precipitates were lysed to detect the intracellular ATP levels. The relative ATP levels were measured with an EnSight multimode plate reader.

### ROS detection.

The levels of ROS in S. aureus ATCC 29213 and E. coli ATCC 25922 treated with IBG alone or with colistin were detected by ROS assay kit (Beyotime, China). Briefly, the cells were washed and resuspended to obtain an OD_600_ of 0.5 with 0.01 M PBS (pH 7.4) and then incubated with dichlorodihydrofluorescein diacetate (DCFH-DA) (10 μM). After 20 min, the cells were treated with different concentrations of IBG for 60 min. The fluorescence intensity of ROS was measured with 488-nm excitation and 525-nm emission filters.

### qRT-PCR study.

The transcript expression levels of *mcr-1* in E. coli SHP45 were determined by qRT-PCR. In brief, a single colony was grown overnight and diluted 1:100 in fresh LB supplemented with various concentrations of IBG (0, 10, 20, 80 μg/mL). After 4 h, total RNA was extracted from the samples by using the TRIzol reagent (Invitrogen, United States), and cDNA was synthesized with the PrimeScript first-strand cDNA synthesis kit (TaKaRa, Japan) according to the manufacturer’s instructions. The qPCR was conducted using the Light Cycler 96 instrument (Roche, Switzerland) with the SYBR premix Ex Taq (TaKaRa, China), and 16S rRNA was used as the endogenous control (Table S5). The correlative expression levels of *mcr-1* were calculated based on the 2^−ΔΔ^*^CT^* method.

### Transcriptome analysis.

E. coli ATCC 25922 cells were grown to an OD_600_ of 0.4 and treated with colistin (0.5 μg/mL) alone or the combination of IBG (40 μg/mL) and colistin (0.5 μg/mL) for 4 h. Samples were collected and preserved with RNAprotect (Qiagen, United States). The total RNA of each sample was extracted using TRIzol reagent (Invitrogen, United States). Control samples were collected from an antibiotic-free culture. RNA sequencing was conducted by the High-Throughput Sequencing Facility at Genewiz, Inc. (Jiangsu, China). Raw sequence data were subjected to quality control using Cutadapt (V1.9.1) and FastQC (V0.10.1). Clean data were aligned with the reference genome of E. coli MG1655 (NCBI accession number NC_000913.3) via the software Bowtie2 (v2.1.0).

### Metabolome analysis.

E. coli for metabolite expression determination was treated using the method used for transcriptome sequencing (RNA-Seq) analysis. Three independent cultures (biological replicates) were performed for all treatments. Intracellular metabolites of E. coli were analyzed by gas chromatography-mass spectrometry (GC-MS). Principal-component analysis (PCA) and partial least-squares-discriminant analysis (PLS-DA) were carried out to evaluate the grouping trends between different treatments. Metabolites with a variable importance in the projection (VIP) value of >1 and a |log_2_ fold change| of >1 were identified as DEMs. KEGG analysis was performed to identify the enriched pathways of DEMs based on previous research.

### G. mellonella infection model.

G. mellonella larvae were obtained from Kaide Ruixin (Tianjin, China). The synergy between IBG and colistin was evaluated in the G. mellonella infection model. The G. mellonella larvae were randomly distributed into five groups (*n* = 10 per group) and infected with 10 μL of E. coli SHP45 suspension (9.8 × 10^4^ CFU) at the right posterior proleg. At 1 h postinfection, G. mellonella were treated with PBS, colistin (2 mg/kg), and IBG alone or combined with colistin (10 mg/kg + 1 mg/kg and 40 mg/kg + 2 mg/kg) at the left posterior proleg. The daily survival of G. mellonella was recorded at 37°C over 5 days.

### Mouse peritonitis-sepsis model.

Six- to 8-week-old female BALB/c mice were used for the mouse peritonitis-sepsis model. Mice were randomly divided into five groups, with six mice per group. Prior to this experiment, mice were acclimated for 5 days in the Laboratory Animal Center of South China Agricultural University. Then, the mice were infected by intraperitoneal injection of 100 μL of 1.8 × 10^8^ CFU E. coli SHP45 suspension or 5.6 × 10^8^ CFU E. coli ATCC 25922 suspension. After 1 h, mice were given PBS, colistin, IBG, and IBG-colistin. The survival rates of treated mice were recorded after 5 days. Once the infected mice died, the liver, spleen, and kidney were removed and subjected to histological analysis.

### Mouse thigh model.

The mouse thigh model was employed for testing the *in vivo* synergistic efficacy of the combination of IBG and colistin. Prior to infection, BALB/c mice were rendered neutropenic by two doses of cyclophosphamide intraperitoneally (150 mg/kg and 100 mg/kg on days 4 and 1, respectively). Mice were infected by injection of a 50-μL suspension of E. coli SHP45 (1.1 × 10^8^ CFU) into the left thigh muscle. At 1 h postinfection, the mice were administered IBG, colistin, and IBG-colistin. After 24 h, mice were sacrificed, and thigh homogenates in sterile normal saline were sampled for bacterial burden quantifications.

### Statistical analysis.

GraphPad Prism 9.0 software was used for statistical analyses. For pairwise comparisons, paired *t* tests were done. The data are expressed as means and standard deviations (SD).

### Ethical approval.

Female BALB/c mice (6 to 8 weeks old; Guangdong Medical Lab Animal Center, Guangzhou, China) were used in this experiment. All animal studies were conducted in accordance with SCAU Institutional Animal Welfare and Ethics guidelines. The animal use procedures were approved by the Animal Research Committees of SCAU (2021b220).

### Data availability.

Source data, including extended data supporting the finding of this study, are provided in the paper. RNA-Seq data have been deposited in the NCBI’s Sequence Read Archive with accession number PRJNA557175.
