# Supplementary material for: Mechanism of Action of Isopropoxy Benzene Guanidine against Multidrug-Resistant Pathogens
Source: Microbiol Spectr. 2022 Dec 8;11(1):e03469-22. doi: 10.1128/spectrum.03469-22 (PMC9927234; doi:10.1128/spectrum.03469-22)
Supplement: Supplemental file 1 — Supplemental material. Download spectrum.03469-22-s0001.pdf, PDF file, 1 MB [file spectrum.03469-22-s0001.pdf]

Isopropoxy Benzene Guanidine Against Multidrug-Resistant Pathogens

Jie Li [a, b, c†], Xiufeng Zhang [a, b, c†], Ning Han [a, b, c], Peng Wan [a, b, c], Feifei Zhao [a, b, c], Tiantian Xu [a, b, c], Xianfeng Peng [d], Wenguang Xiong [a, b, c], Zhenling Zeng [a, b, c] *

a Guangdong Provincial Key Laboratory of Veterinary Pharmaceutics Development and Safety Evaluation, College of Veterinary Medicine, South China Agricultural University, Guangzhou 510642, China.
b National Laboratory of Safety Evaluation (Environmental Assessment) of Veterinary Drugs, South China Agricultural University, Guangzhou 510642, China.
c National Risk Assessment Laboratory for Antimicrobial Resistance of Animal Original Bacteria, South China Agricultural University, Guangzhou 510642, China.
d Guangzhou Insighter Biotechnology Co., Ltd, Guangzhou 510642, China

† These authors contribute equally.

*Corresponding author.

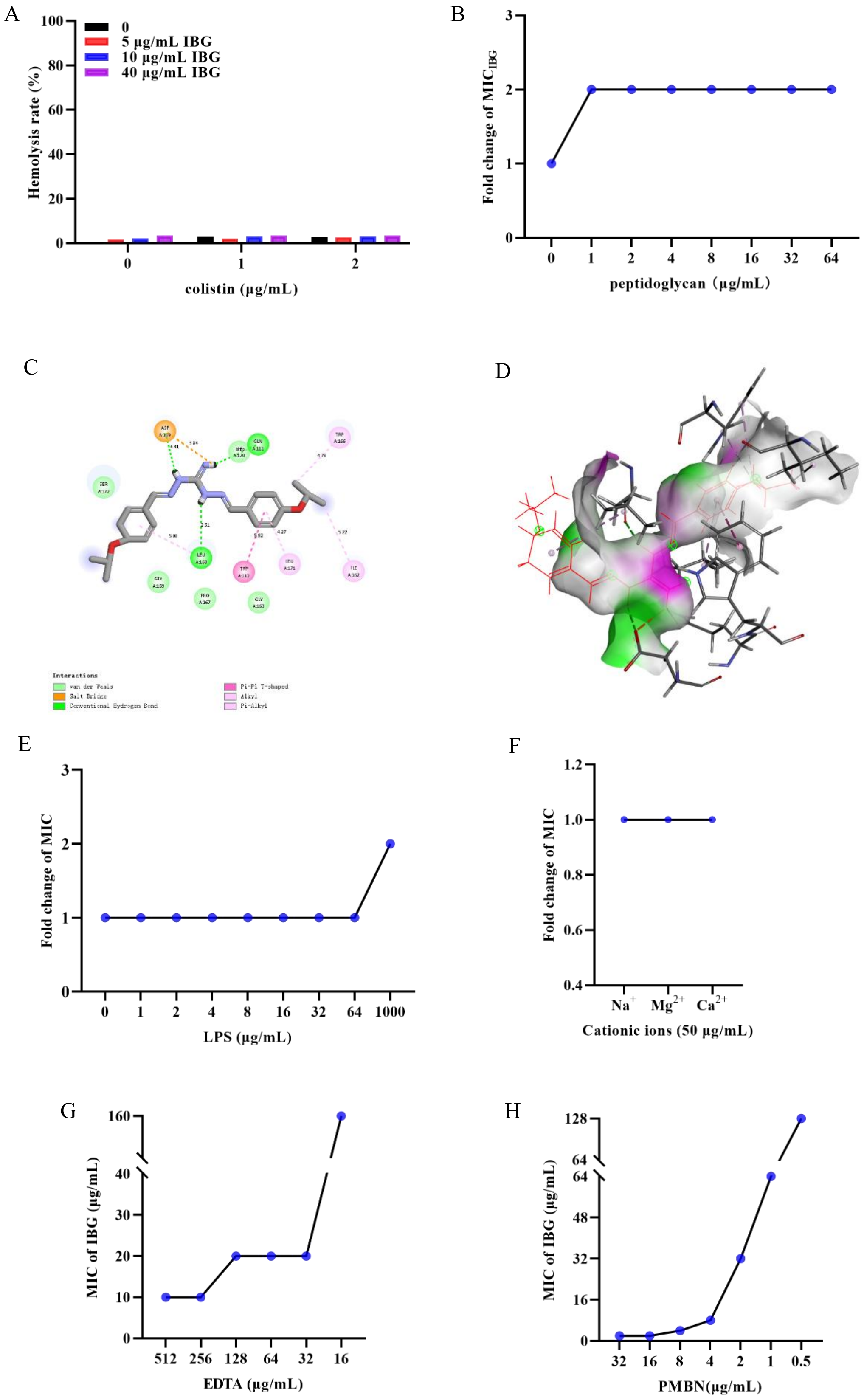
A
0
5 μg/mL IBG
10 μg/mL IBG
40 μg/mL IBG
Hemolysis rate (%)
100
80
60
40
20
0
0
1
2
colistin (μg/mL)
B
Fold change of MIC$_{IBG}$
3
2
1
0
0
1
2
4
8
16
32
64
peptidoglycan（μg/mL）
C
Interactions
van der Waals
Salt Bridge
Conventional Hydrogen Bond
Pi-Pi T-shaped
Alkyl
Pi-Alkyl
D
E
Fold change of MIC
3
2
1
0
0
1
2
4
8
16
32
64
1000
LPS (μg/mL)
F
Fold change of MIC
1.2
1.0
0.8
0.6
0.4
Na^+
Mg^{2+}
Ca^{2+}
Cationic ions (50 μg/mL)
G
MIC of IBG (μg/mL)
160
40
30
20
10
0
512
256
128
64
32
16
EDTA (μg/mL)
H
MIC of IBG (μg/mL)
128
64
64
48
32
16
0
32
16
8
4
2
1
0.5
PMBN(μg/mL)

I

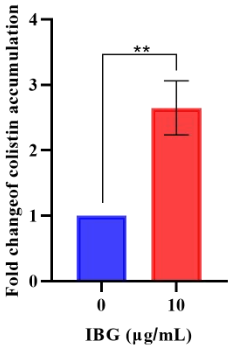

J

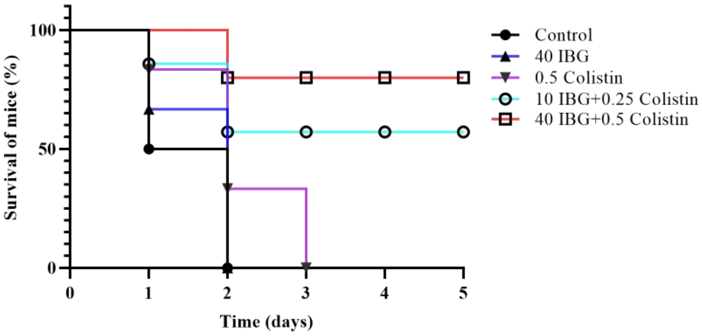

Figure S1 The antibacterial effects of IBG against multidrug-resistant bacteria.

(A) The hemolytic activity of colistin (0, 1, 2 μg/mL) in the absence or presence of IBG on sheep red blood cells.

(B) Exogenous addition of peptidoglycan from *S. aureus* impairs the antibacterial activities of IBG slightly against *S. aureus* ATCC 29213 determined by chequerboard microdilution assay.

(C, D) 2D molecular interactions (C) and 3D molecular interactions (D) of IBG and pgsA was assessed by receptor-ligand interaction analysis.

(E) The change in MIC of IBG against *S. aureus* ATCC 29213 in the presence of lipopolysaccharide (LPS) ranges from 0 to 1 mg/mL. High levels of LPS (1 mg/mL) impair the antibacterial activity of IBG slightly.

(F) The change in MIC of IBG against *S. aureus* ATCC 29213 in the presence of 50 μg/mL different cations. Divalent cations had a neglectable influence on the antibacterial activity of IBG.

(G, H) A synergy of IBG with the EDTA (G) and PMBN (H) against *E. coli* ATCC25922 by checkerboard microdilution. PMBN and EDTA could increase the antibacterial activity of IBG in a dose-dependent manner.

(I) Effect of IBG on the accumulation of colistin in *E. coli* SHP45. Data were presented as means ±SD (n = 3 biological independent replicates) and t-test were used to calculate P-values (*$p < 0.05$, **$p < 0.01$).

(J) Survival rates of mice were infected with colistin-susceptible *E. coli* ATCC25922 in the mouse peritonitis–sepsis model.

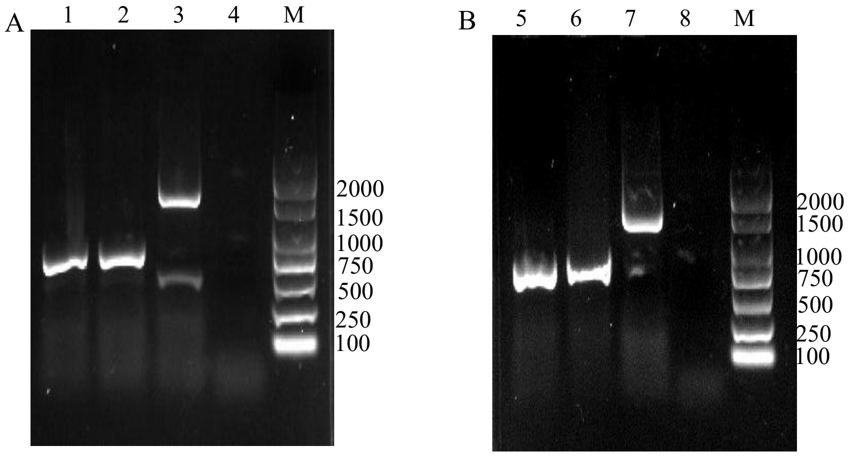

Figure S2 Identification of the *E. coli* LPS deletion strains MG1655-Δ*waaC* (A) and MG1655-Δ*waaP* (B).

M: Standard DNA marker (DL-2000); 4, 8: Negative control; 1, 2: MG1655-Δ*waaC*; 5, 6: MG1655-Δ*waaP*; 3, 7: *E. coli* MG1655

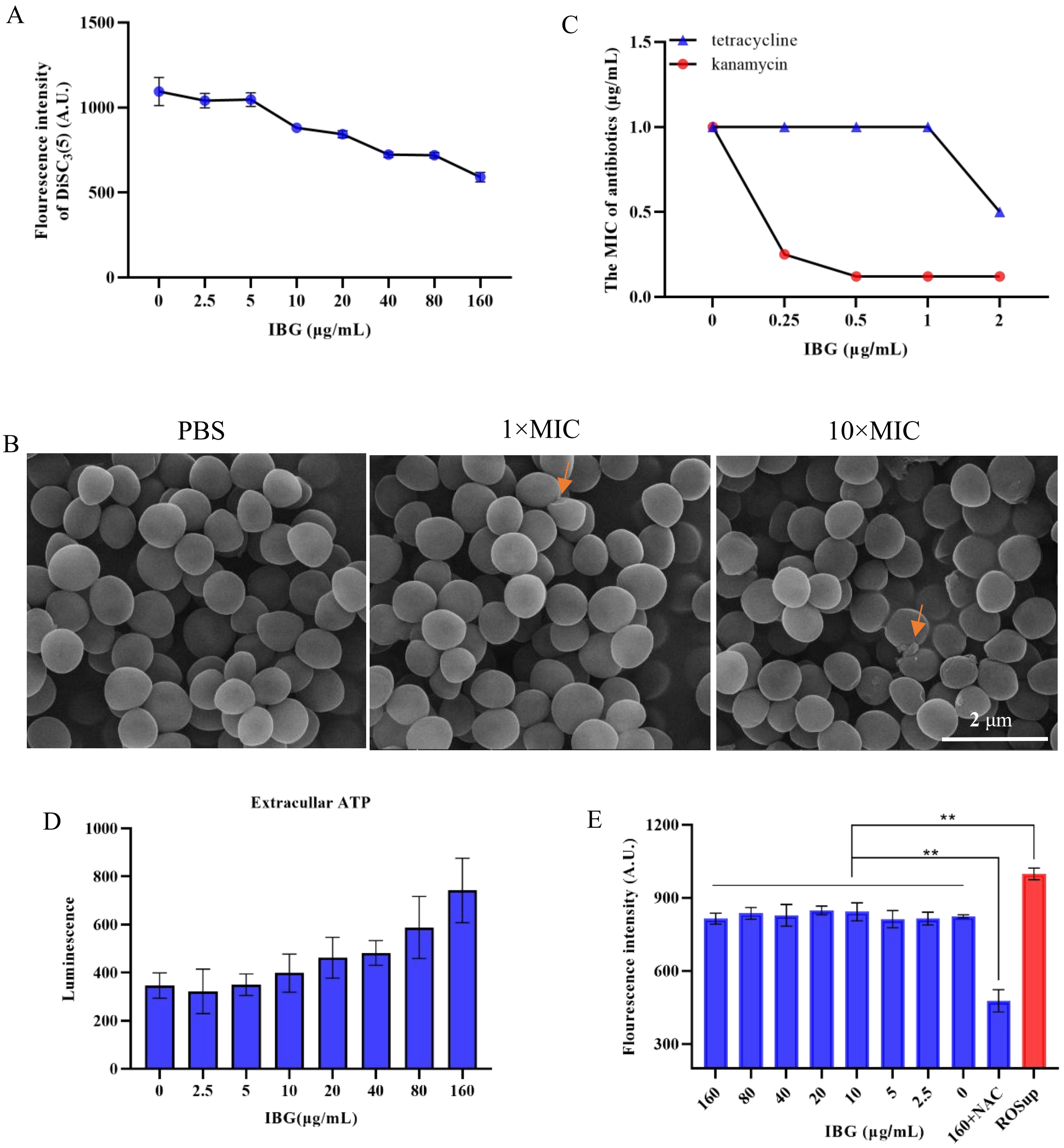

Figure S3 Mechanism of IBG against Gram-positive bacteria

(A) Decreased the fluorescence of membrane potential in *S. aureus* ATCC 29213 treated with IBG. Membrane potential of the inner membrane was detected by DiSC$_3$(5).

(B) Morphological changes of *S. aureus* ATCC 29213 incubated with various doses of IBG were imaged by FE-SEM, Scar bar, 2 μm. Bacteria without treated as control. The red arrows represent bacteria with destroyed and lysed structure.

(C) IBG is synergistic with kanamycin, and no synergy combined with tetracycline against *S.*

aureus ATCC 29213.

(D) Decreased levels of extracellular ATP in *S. aureus* ATCC 29213 after treatment of IBG.

(E) IBG had a neglectable influence on the accumulation of reactive oxygen species (ROS) against *S. aureus* ATCC 29213. Data points represent the mean value of three biological replicates, P-values was calculated by nonparametric one-way ANOVA (*$p < 0.05$, **$p < 0.01$).

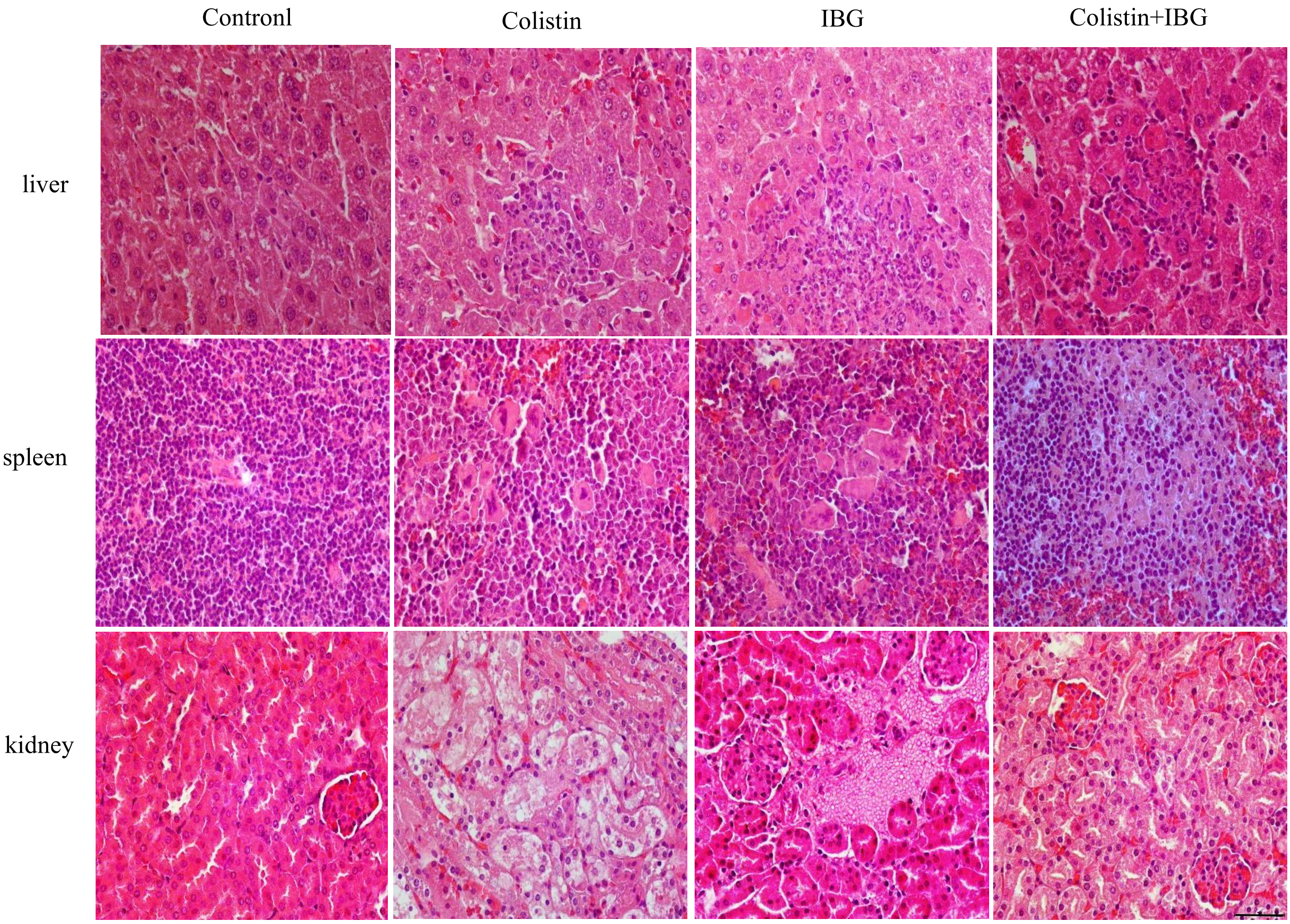

Figure S4 Histologic analysis of different organs using hematoxylin-eosin (HE) staining
The liver, spleen, and kidney were histological analysis in the mouse peritonitis-sepsis model. The results were representative of three biological repeats. Scar bar, 50 μm.

Table S1 FICI of combinations of IBG and other antimicrobial agents against *E. coli*

Combination	FICI		Effect
	ATCC 25922	SHP 45	
IBG+ polymyxin B	0.12	0.24	Synergy
IBG+ colistin	0.12	0.12	Synergy
IBG+ trimethoprim	0.6	0.6	Addition
IBG+ ceftazidime	2	2	Indifference
IBG+ amoxicillin	2	2	Indifference
IBG+ cefotaxime	2	2	Indifference
IBG+ cefoxitin	2	2	Indifference
IBG+ meropenem	2	2	Indifference
IBG+ tetracycline	2	2	Indifference
IBG+ amikacin	2	2	Indifference
IBG+ ciprofloxacin	2	2	Indifference
IBG+ streptomycin	2	2	Indifference
IBG+ chloramphenicol	2	2	Indifference
IBG+ florfenicol	2	2	Indifference
IBG+ fosfomycin	2	2	Indifference
IBG+ neomycin	2	2	Indifference

Table S2 All Strains treated with the 10 μg/mL IBG combined with 0.25 μg/mL colistin

strains	character	strains	character
E. coli ATCC25922	reference strain	*k. pneumoniae* K52-2	Wildtype
E. coli SHP45	*mcr-1*	*k. pneumoniae* K35	Wildtype
E. coli GDQ20D140	*mcr-1*	*k. pneumoniae* K107-2	Wildtype
E. coli GDQ8D137	*mcr-1*	*K. pneumoniae* 117	*mcr-1*
E. coli GDQ8D43	*mcr-1*	*A. baumannii* ATCC19606	reference strain
E. coli GDQ8D105	*mcr-1*	*A. baumannii* 131312	Wildtype
E. coli GDQ8P37	*mcr-1*	*A. baumannii* 130939	Wildtype
Salmonella ATCC14028	reference strain	*A. baumannii* 131172	Wildtype
Salmonella 26FS14	*mcr-1*	*P. muhocida* CVCC434	reference strain
Salmonella S226	*mcr-1*	*P. muhocida* 18	Wildtype
Salmonella S235	*mcr-1*	*P. muhocida* 23	Wildtype
Salmonella F18126S	*mcr-1*	*P. multocida* 89	Wildtype
Salmonella F19062S	Wildtype	*P. multocida* 117	Wildtype
Salmonella F19139S	Wildtype	*Salmonella* F19112S	Wildtype
Salmonella F19069S	Wildtype		

Table S3 The MIC of IBG against *E. coli* LPS mutant bacteria

Organism	MIC (μg/mL)		FICI
	IBG	Colistin	
MG1655	＞256	0.5	0.06
MG1655-Δ*waaC*	4	0.12	0.625
MG1655-Δ*waaP*	4	0.12	0.75

Table S4 MRM parameters for the determination of colistin by LC-MS/MS

**Compound**	**Precursorion （m/z）**	**Product ions （m/z）**	**Q1 pre bia (V)**	**Collision energy (eV)**	**Q3 pre bias (V)**
Colistin A	**390.7**	**101.1**	**29**	**21**	**11**
		**384.8***	**16**	**13**	**29**
Colistin B	385.95	101.1*	10	22	28
		379.9	28	13	21

Table S5 Bacterial strains used in this study

strains	character	strains	character
S. aureus ATCC 29213	reference strain	*K. pneumoniae* K52-2	Wildtype
E. faecalis ATCC 29212	reference strain	*K. pneumoniae* K35	Wildtype
E. coli ATCC 25922	reference strain	*K. pneumoniae* K107-2	Wildtype
E. coli SHP45	*mcr-1*	*K. pneumoniae* *MPC11*＋pHNSHP45	*mcr-1*
E. coli GDQ20D140	*mcr-1*	*K. pneumoniae* *MPC11*	Wildtype
E. coli GDQ8D137	*mcr-1*	*K. pneumoniae* 117	*mcr-1*
E. coli GDQ8D43	*mcr-1*	*K. pneumoniae*281	*mcr-1*
E. coli GDQ8D105	*mcr-1*	*A. baumannii* ATCC19606	reference strain
E. coli GDQ8P37	*mcr-1*	*A. baumannii* 131312	Wildtype
Salmonella F19112S	Wildtype	*A. baumannii* 130939	Wildtype
Salmonella ATCC14028	reference strain	*A. baumannii* 131284	Wildtype
Salmonella 26FS14	*mcr-1*	*A. baumannii* 131172	Wildtype
Salmonella S226	*mcr-1*	*P. muhocida* CVCC434	reference strain
Salmonella S235	*mcr-1*	*P. muhocida* CVCC 399	reference strain
Salmonella F18126S	*mcr-1*	*P. muhocida* 18	Wildtype
Salmonella F19062S	Wildtype	*P. muhocida* 23	Wildtype
Salmonella F19139S	Wildtype	*P. muhocida* 89	Wildtype
Salmonella F19069S	Wildtype	*P. muhocida* 117	Wildtype
Salmonella F19112S	Wildtype	*K. pneumoniae* ATCC 700603	reference strain

Table S6 Primers used in this study

Primer names	Sequence (5' to 3')	Product size（bp）
pEcgRNA -F	TTTTGTGATGCTCGTCAGGGGG	643
pEcgRNA -R	CGCTCGATGACGCCAACTACCT	
waaC-F1	TTTGAATACCGAGCAGCAGGCA	349
waaC-F2	ATGTTAGCATGTTTTACCTCCGTCAGGCTTCCTCTTGT	
waaC-F3	ACAAGAGGAAGCCTGACGGAGGTAAAACATGCTAACAT	324
waaC-F4	ACTATGGTAAGTAGCACGAAATGG	
waaCtest-F	GATTAAGTTTGACTGGGTGGT	756
waaCtest-R	GTTGGTCCATAAACCGTGATA	
waaCspacer-F	TAGTGGGGTTAAGCCATTTAACGG	--
waaCspacer-R	AAACCCGTTAAATGGCTTAACCCC	
waaP-F1	ATCTTCGCCAACAGTATTTCTTAG	345
waaP-F2	TCATTACAGGTGGTTTAGAAATATTTATTAGTATGTAC	
waaP-F3	GTACATACTAATAAATATTTCTAAACCACCTGTAATGA	311
waaP-F4	AGCCGCTGATTTATTACTGC	
waaPtest-F	GTTCGCTCTTTAATACGCTC	748
waaPtest-R	TTTGGCGTGGTAAAGATGCT	
waaPspacer-F	TAGTAGTGGCAAATGTAACAGTCG	--
waaPspacer-R	AAACCGACTGTTACATTTGCCACT	
mcr-1-RTPCR-F	AAAGACGCGGTACAAGCAAC	213
mcr-1-RTPCR-R	GCTGAACATACACGGCACAG	
16s-RTPCR-F	TCCTACGGGAGGCAGCAGT	467
16s-RTPCR-R	GGACTACCAGGGTATCTAATCCTGTT	
